# Institutional Notes and News

**Published:** 1910-09-24

**Authors:** 


### Institutional Notes and News.

**General Infirmary, Worcester.**

At the meeting of the Executive Committee some discussion took place on the subject of contributions from patients whose means permitted them to make some payment. This arose over a payment of £10 10*s.* received from a former patient who had been admitted suffering from a severe accident, and who had been an inmate for twenty-one weeks. It was finally decided that a letter be written showing the straitened conditions of the finances of the institution, and asking for a larger donation. The committee, however, agreed that the principle of making a definite charge for treatment could not be accepted.

**Bristol General Hospital.**

The work done at the Bristol General Infirmary continues to grow, as the increase of 1,000 cases treated at the out-patient department, mentioned at the recent half-yearly meeting of governors, presided over by Mr. J. Storrs Fry, proves eloquently. The beds are full, and the in-patient department can care for no more cases unless the present accommodation is increased. The pressure is greatest in the female medical and surgical wards, and if the public will only come forward and provide the money it is hoped to extend these wards, and, if possible, to increase also the administration buildings. A maternity ward, too, is badly needed. The dental department, let us add, which is situated now in two houses beyond the hospital, could be run more smoothly, and therefore efficiently, if it, too, formed part of the institution. Mr. C. B. Hare (vice-president) supported Mr. Storrs Fry's contentions by giving a general account of the work and financial prospects of the year. The plan of raising further money to carry out the extensions and improvements above outlined is hampered, it seems, by the public superstition that the hospital is a very wealthy one. As Mr. Hare pointed out, one can hardly call an institution very wealthy when its overdraft approaches £5,000, and, in spite of economies, by Christmas, he feared, will reach that amount. Mrs. Proctor Baker has started the fund with £5,000 and what the Bristol public might be reminded of very well, and quite truly, is that Bristol is a very wealthy city. A general hospital with 180 beds that are occupied to the full has a record of work behind it which no city, much more no wealthy city, can forget. We hope that Mrs. Proctor Baker's generous example will be imitated by others in Bristol.

**York County Hospital.**

We take the following from the quarterly report of the House Committee of York County Hospital just issued: "During the past quarter one of the most important efforts ever promoted on behalf of the hospital funds has been brought to a successful issue by the raising of upwards of £2,000 in special donations to meet the offer of £1,000 from Messrs. H. H. and F. Riley-Smith. . . . The Committee finds it difficult adequately to express its gratitude to those who have so willingly come forward with their contributions at a time when the hospital was much in need of their help." Alderman Sir Joseph Sykes pointed out that only an increased income could prevent a recurrence of the debt, which had been cleared by the public spirit of the neighbourhood. He remarked also, in response to a question, that he was not aware that the Charity Commissioners proposed any scheme for the amalgamation of the city charities which would affect the hospital; but were this done the hospital must be properly represented on any committee that was formed to discuss it. A vote hoping that the City Council would decline to draft any scheme to rob the city of the control of its charities was proposed by Mr. W. Hargrave, one of the York Charity Trustees, and carried unanimously.

**Future of Whitgift Hospital.**

The future of the historic Whitgift Hospital has been exercising the neighbourhood of Croydon for some months past, and indeed that section of the general public which supports the Society for the Protection of Ancient Buildings and other bodies has been watching jealously the doom that has been threatening it. Last week the Croydon Borough Council again discussed it, and by 38 votes to 13 decided to apply to the Local Government Board for a provisional order containing powers to acquire some 30 properties, whereby the new frontage line would be continued to within about 70 feet of these Elizabethan almshouses. The Streets Improvement Committee gained the support of the meeting for the above application by arguing that it was that part of the old scheme which had been regarded always as non-controversial. The hospital, said the Committee, would not be endangered by it, because it reached a point where the alternative scheme for widening could be begun afterwards. The substantial majority which supported these contentions shows that these arguments were felt generally to be sound, and that Whitgift Hospital is safe from destruction, at any rate for the moment.

**Wallsal and District Hospital.**

For the past eighteen months a representative committee of the townspeople of Walsall have been hard at work organising a grand bazaar in aid of the funds of the hospital, and judging by the enthusiastic manner in which the movement has been taken up, there seems but little doubt that the object of the committee, to raise a sum of £3,000, will be fully realised. The Bazaar, which will be entitled the "Historic" Bazaar—each stall representing a different period in the world's history—will be opened on November 1 by the Countess of Bradford, and on the following day by the Countess of Lichfield. One of the principal items of a very attractive programme of entertainment to be given, will be the Blue Hungarian Band. The funds realised will be devoted to clearing off a debt of £2,049 on the maintenance account of the hospital, and to relieving the stress which lack of current means has given rise to, by re-opening some of the new beds which were closed two years ago. Continuous efforts have been made during the past two years, and recently special steps have been taken by the Committee to permanently increase the income of the Institution. The hospital has recently benefited to the extent of £1,000 under the will of the late Frederick Birch, of Walsall.

---

Over three thousand applications for appointments in the new infirmary of the Wandsworth Union have been submitted to the Guardians. Mr. W. L. MacCormac, who was senior assistant medical superintendent of the old infirmary, has been appointed medical superintendent of the new institution, with Dr. C. C. Neates, now junior medical assistant at the old infirmary, as senior, and Mr. H. B. Morgan as junior assistant. The chaplaincy, for which there were forty-nine applications, has been conferred on the Rev. H. E. Whittington, curate of Hornsey Parish Church.

